# MicroRNA Signature in Alcoholic Liver Disease

**DOI:** 10.1155/2012/498232

**Published:** 2012-01-30

**Authors:** Shashi Bala, Gyongyi Szabo

**Affiliations:** Department of Medicine, University of Massachusetts Medical School, Worcester, MA 01605, USA

## Abstract

Alcoholic liver disease (ALD) is a major global health problem. Chronic alcohol use results in inflammation and fatty liver, and in some cases, it leads to fibrosis and cirrhosis or hepatocellular carcinoma. Increased proinflammatory cytokines, particularly TNF alpha, play a central role in the pathogenesis of ALD. TNF alpha is tightly regulated at transcriptional and posttranscriptional levels. Recently, microRNAs (miRNAs) have been shown to modulate gene functions. The role of miRNAs in ALD is getting attention, and recent studies suggest that alcohol modulates miRNAs. Recently, we showed that alcohol induces miR-155 expression both in vitro (RAW 264.7 macrophage) and in vivo (Kupffer cells, KCs of alcohol-fed mice). Induction of miR-155 contributed to increased TNF alpha production and to the sensitization of KCs to produce more TNF alpha in response to LPS. In this paper, we summarize the current knowledge of miRNAs in ALD and also report increased expression of miR-155 and miR-132 in the total liver as well as in isolated hepatocytes and KCs of alcohol-fed mice. Our novel finding of the alcohol-induced increase of miRNAs in hepatocytes and KCs after alcohol feeding provides further insight into the evolving knowledge regarding the role of miRNAs in ALD.

## 1. Introduction

MicroRNAs (miRNAs) are 20–22 nucleotides long noncoding RNAs that were first described in 1993 [1]. MiRNAs play a central role in diverse cellular processes including development, immunity, cell-cycle control, metabolism, viral or bacterial disease, stem-cell differentiation, and oncogenesis [2–4]. In general, miRNAs are transcribed from RNA polymerase II or III in the nucleus and transported to the cytoplasm, where they are processed into mature miRNAs [4]. Mature miRNAs can target hundreds of genes by either binding to the 3′ or 5′ untranslated (UTR) region of mRNA [4]. Emerging evidence suggests that miRNAs not only target mRNAs but also they are capable of modulating transcription and methylation processes [5–7]. Moreover, not only the sense strand (miRNA) of mature miRNA modulates gene function, but also the antisense strand (star form; *) plays an important role in the miRNA regulatory network [8]. However, the biological significance of the antisense strand (star form) is largely unknown but is slowly getting attention. In a short time, miRNA research has received tremendous attention due to their fine-tuning roles in almost all biological pathways. Moreover, disease-specific tissue miRNA signatures have been identified in various etiologies such as hepatocellular carcinoma (HCC), hepatitis C virus (HCV), hepatitis B virus (HBV), cardiac disease, neuroinflammation, rheumatic arthritis (RA), and various cancers [3, 9–14]. In this paper, we highlight the emerging roles of miRNAs in alcoholic liver disease.

### 1.1. MiRNA in Innate Immune Response

Innate immunity is the first line of host defense against foreign pathogens and also in response to damaged self (endogenous danger signals). Toll-like receptors (TLRs) are the most widely studied danger signal sensors. MiRNAs have been implicated in various immune responses and are believed to be essential regulators of these processes [15]. The number of miRNAs involved in immune responses is growing, and among them, miR-155, -146a, -125b, -132, -9, -212 and -181, are the key players and are elegantly reviewed in [16, 17]. The inflammation-related miRNAs deserve attention in ALD, as the activation of the innate immune system is a hallmark of alcoholic steatohepatitis.

## 2. MiRNA in Alcoholic Liver Disease

### 2.1. Alcoholic Liver Disease (ALD)

Alcoholic liver disease (ALD) is a global health-related problem, which contributes significantly to liver-related mortality. Increased inflammation and fat accumulation are the hallmarks of ALD. The progression of ALD involves a complex network of signaling molecules and chronic alcohol abuse in some cases leads to liver cirrhosis [18]. Alcohol alone or its metabolites (acetaldehyde) act on multiple signaling pathways and result in increased intestine permeability and ROS generation [19, 20]. Increased gut permeability is associated with translocation of bacteria and bacterial products into the lumen of the intestine, which results in the imbalance of intestine homeostasis [20]. LPS is a major component of a Gram-negative bacterial cell wall, and it is detoxified in the liver via both parenchymal and nonparenchymal cells [21, 22]. It is believed that increased LPS in the circulation disrupts the liver homeostasis, resulting in Kupffer cell (KC; liver macrophages) activation. Upon activation, KCs produce TNF alpha, which then induces the activation of other signaling cascades to amplify the inflammation. TNF alpha-induced inflammation is more prevalent in alcoholic hepatitis [23]. The role of the LPS/TLR4 axis has been appreciated in ALD, since TLR4 KO mice have been shown to be protected from liver damage in a mouse model of ALD [24].

### 2.2. MiRNA Profiling in the Livers of Alcohol-Fed Mice

Alcohol has been shown to modulate the epigenetic factors in various organs including liver and brain and was reviewed recently [25]. As alcohol exerts epigenetic effects, it is conceivable that alcohol might target miRNAs to regulate gene functions. To date, there are very few studies related to the roles of miRNAs in ALD. Previously, our laboratory demonstrated the differential expression of some miRNAs in the livers of alcohol-fed mice by microarray analysis [26]. MiR-27b, miR-214, miR-199a-3p, miR-182, miR-183, miR-200a, and miR-322 were found to be downregulated, whereas miR-705 and miR-1224 were increased after 4 weeks of alcohol feeding in mice [26]. However, the physiological relevance of these miRNAs in ALD has yet to be determined.

### 2.3. The Role of miRNA in Alcohol-Induced Intestinal Permeability

Alcohol and its metabolites are known to increase intestinal permeability [27]. In the past, various signaling molecules and transcription factors were reported to be involved in alcohol-mediated intestinal permeability. Recently, miR-212 has been identified as a new player and implicated in alcohol-induced intestinal permeability, where it targets a major tight junction protein, Zonula occludens 1 (ZO-1) [28]. ZO-1 plays an essential role in the regulation of intestinal permeability. Induction of miR-212 and decrease in ZO-1 protein were observed both in colon biopsy samples from patients with ALD and in alcohol-treated CaCO-2 cells [28].

 Recently, miR-29a and miR-122a were reported to modulate intestinal membrane permeability. MiR-29a regulates intestinal membrane permeability in patients with irritable bowel syndrome (IBS) [29]. Increased expression of miR-29a was found in blood microvesicles, small bowel, and colon tissues of IBS patients. MiR-29a targets the glutamine synthetase gene (GLUL), which in turn regulates intestinal membrane permeability [29]. MiR-122a was found to target occludin (a transmembrane tight junction protein) both in Caco-2 cells and mice enterocytes and hence plays an important role in regulating intestinal permeability [30]. The potential role of these miRNAs in alcohol-induced intestinal permeability is yet to be determined.

### 2.4. Potential Role of miRNA in Alcohol-Mediated Oxidative Stress

Not only does increased circulating endotoxin play a crucial role in ALD, but also increased reactive oxygen species (ROS and oxidative stress) production contributes to the pathogenesis of ALD [20, 27]. In agreement with this, NADPH oxidase is reported as a major source of oxidants in ALD, and mice deficient in this oxidase (p47^phox−/−^) were protected from early alcohol-induced liver injury [31]. In general, the potential role of miRNAs in oxidative stress-mediated etiologies is emerging. In line with this, miR-27a*, miR-27b*, miR-29b*, miR-24-2*, and miR-21* were reported to be differentially expressed in response to H_2_O_2_-induced oxidative stress in RAW 264.7 macrophage [32]. Furthermore, overexpression of miR-27b* suppressed LPS-induced activation of NF-*κ*B [32]. These data suggest that macrophage function can be regulated by oxidative stress-responsive miRNAs via modulating the NF-*κ*B pathway [32]. Interestingly, in this study, only the star form of mature miRNA was found to play a role in H_2_O_2_-induced oxidative stress. To our knowledge, there are no studies indicating the direct involvement of miRNAs in alcohol-induced oxidative stress.

 Alcohol has been shown to downregulate miR-199 in rat liver sinusoidal endothelial cells (LSECs) and human endothelial cells [33]. Decreased miR-199 was associated with increased mRNA expression of endothelin-1 (ET-1) and hypoxia-inducible factor-1*α* (HIF-1*α*) [33]. Authors concluded that alcohol-induced ET-1 likely contributes to inflammation in patients with cirrhosis.

### 2.5. Role of miRNA-155 in Kupffer Cell Activation in Alcoholic Liver Disease

Previous studies from our laboratory and others have shown increased TNF alpha in vivo and in vitro alcohol models [34, 35]. Moreover, increased TNF alpha was found in patients with alcoholic hepatitis [23]. Previously, alcohol has been shown to regulate TNF alpha mRNA stability in RAW 264.7 macrophage and KCs [35]. Furthermore, miRNAs modulate gene expression both at posttranscriptional and translational levels [3, 5, 6]. Recently, we have shown that prolonged alcohol exposure induces miR-155 in RAW264.7 macrophage and KCs [34]. However, no changes were found in the expression of miR-146a and -125b, which are also involved in immune responses. This observation suggests that alcohol particularly targets miR-155 [34]. We further showed that miR-155 expression correlates with TNF alpha levels. Functionally, miR-155 regulates TNF alpha mRNA stability, and thereby contributes to increased TNF alpha in KCs of alcohol-fed mice. TNF alpha is tightly regulated both at transcriptional and posttranscriptional levels. Posttranscriptional regulation of TNF alpha includes mRNA stability, polyadenylation, and translational initiation, which target adenine and uridine- (AU-) rich elements (AREs) in its 3UTR [36]. Trans-acting factors such as HuR, tristetraprolin (TTP), and TIA-1, bind to 3UTR of TNF alpha and modulate its expression either by stabilizing or destabilizing its transcripts [36]. Interestingly, chronic alcohol has been shown to increase TNF alpha mRNA stability via HuR protein [35, 37, 38]. As our results indicated that miR-155 enhances TNF alpha transcript stability, it is plausible to suggest that miR-155 might be exerting its effect via targeting HuR or other trans-acting factors. Hence, our study directly or indirectly implicates the role of miR-155 in TNF alpha regulation. Given the fact that one miRNA can regulate multiple genes of a pathway; it is likely that miR-155 might regulate other genes, which are directly or indirectly involved in TNF alpha regulation.

### 2.6. Induction of miRNA in the Livers of Alcohol-Fed Mice

As described above, previously, we found induction of miR-155 in alcohol-exposed RAW264.7 macrophage and KCs of alcohol-fed animals [34]. Here, we examined the liver expression of other miRNA including miR-132, -125b, and -146a which also regulate various immune responses. MiR-132 was shown to play a role in neuroinflammation [39] and also regulates innate antiviral immunity, where it targets the p300 transcriptional coactivator [40]. However, the role of miR-132 in the alcohol-mediated TLR response is yet to be elucidated; therefore, in this study, we determined the effect of alcohol on miR-132. MiR-146a is linked with endotoxin tolerance, where it regulates IRAK-1 and TRAF-6 and acts as a negative regulator of TLR4 signaling [41], whereas miR-125b limits TNF alpha production in RAW 264.7 macrophage [42].

**Figure 1:**
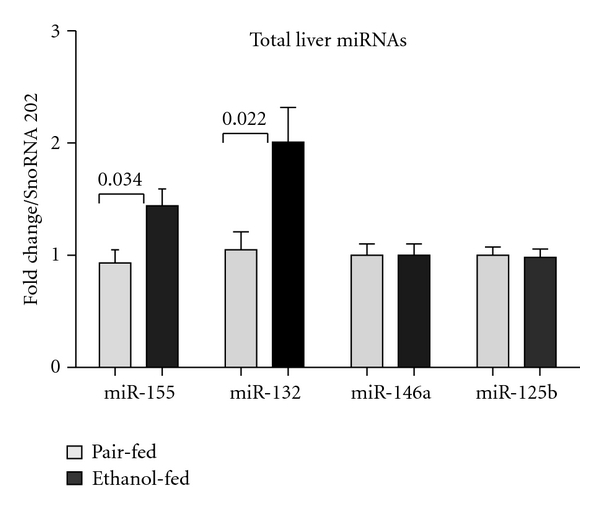
Increased expression of miR-155 and miR-132 in the livers of alcohol-fed mice. C57BL/6 eight-week-old female mice (*n* = 6/group) were fed with Lieber-Decarli diet either containing 5% alcohol (ethanol-fed) or isocaloric liquid diet (pair-fed) for 4 weeks. After 4 weeks, the livers were isolated and stored in RNA later (Qiagen) for RNA analysis at −80°C. Total RNA from livers was isolated using the miRNeasy kit (Qiagen), and quantification of miRNAs was carried out by TaqMan miRNA assays (Applied Biosystems). The data were normalized to SnoRNA202 (endogenous control) and shown as fold change over the pair-fed control group. Data represent mean values ± S.E.M. Statistical significance was determined using T-test (two-tailed).

 Among the miRNAs tested, we found significant induction of miR-132 in the livers of alcohol-fed mice (Figure 1). As expected, a significant increase in miR-155 was also observed in the livers of alcohol-fed mice (Figure 1), and this result was consistent with our previous report [21]. Contrary to this observation, no significant changes were observed in miR-125b and -146a expression (Figure 1). The mice used in the study were housed and cared for as per animal protocols approved by the Institutional Animal Use and Care Committee of the University of Massachusetts Medical School.

### 2.7. Increase in miR-155 in Hepatocytes of Alcohol-Fed Mice

Because of the crucial role of miR-155 in inflammation, most studies are focused on its role in immune cells such as monocytes, macrophages, dendritic cells, and T cells (reviewed in [16, 17, 41]). Induction of miR-155 has been reported in various inflammatory-related diseases such as RA, neuro- or autoimmune-inflammation and various cancers [43–46]. The concept of cell-specific effects of miRNA is emerging, and reports showing the role of immune-related miRNAs in hepatocytes are sparse. Recent studies suggest that hepatocytes do respond to LPS-induced TLR signaling and express functional NOD1 and 2 receptors [47]. Therefore, next, we examined the effect of alcohol on miRNA expression in hepatocytes, which are the predominant liver-cell population.

 Hepatocytes were isolated from mice fed with Lieber-DeCalri diet either with 5% alcohol (ethanol-fed) or isocaloric diet (pair-fed) for 4 weeks [34]. Hepatocytes isolation was performed with the method described earlier by our group [24]. Total RNA was isolated with the miRNeasy kit (Qiagen) and subjected to miRNA analysis as described previously [34]. Briefly, TaqMan miRNA assay (Applied Biosystems) was used, and snoRNA202 was used as internal control to normalize the technical variations between the samples. Fold change was calculated in comparison to pair-fed mice.

**Figure 2:**
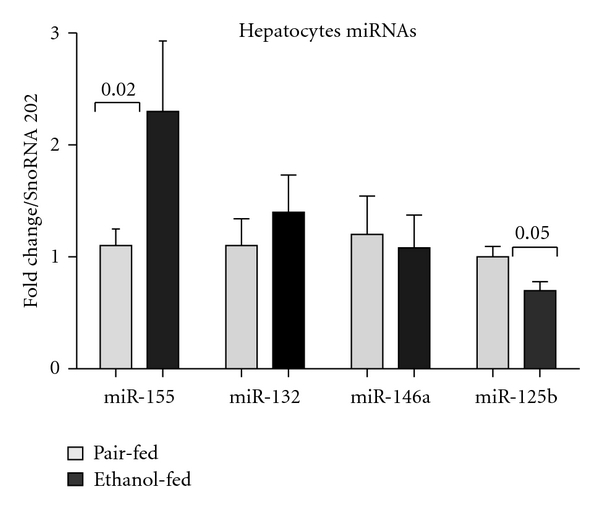
Induction of miR-155 in hepatocytes of alcohol-fed mice. Hepatocytes were isolated from the mice (*n* = 5-6) after 4 weeks of feeding. Briefly, the livers were perfused, followed by digestion. Parenchymal cells (hepatocytes) were collected after low-speed centrifugation, washed, and were plated onto 6-well collagen-coated plates (BD-Biosciences). After 3 h, floating cells were removed, and adherent cells were washed twice with PBS and lysed in QIAzole (Qiagen). Total RNA was isolated and miRNA expression was quantified by TaqMan miRNA assays (Applied Biosystems). The data were normalized to SnoRNA202 (endogenous control) and shown as the fold change over the pair-fed control group. Data represent mean values ± S.E.M. Statistical significance was determined using non-parametric Mann-Whitney test.

 Interestingly, we found increased expression of miR-155 in hepatocytes of alcohol-fed mice compared to pair-fed mice (Figure 2). To our best knowledge, this is the first study reporting the induction of miR-155 in hepatocytes after alcohol feeding. No obvious changes were observed in miR-146a expression and there was minimal increase in miR-132 expression in hepatocytes of alcohol-fed mice (Figure 2). In contrast, expression of miR-125b was found to be downregulated in hepatocytes after alcohol feeding (Figure 2). The physiological relevance of alcohol-induced miR-155 in hepatocytes is the subject of ongoing investigation.

 In diet-induced (methyl-deficient diet) nonalcoholic steaohepatitis, increased miR-155 was associated with decreased levels of C/EBP-*β* and SOCS1 proteins [48]. In vitro overexpression of miR-155 in mouse primary hepatocytes resulted in a decreased level of C/EBP-*β* and SOCS1 proteins [48]. Both C/EBP-*β* and SOCS1 are tumor suppressors [49] and often downregulated in hepatocellular carcinomas and hepatoblastomas [50]. The relevance of C/EBP-*β* and SOCS1 could be translated to ALD due to the fact that SOCS1 not only acts as a tumor suppressor, but also is a negative regulator of LPS signaling, while chronic alcohol use results in increased inflammatory cytokine production. Moreover, SOCS1 is also an important mediator of cellular oxidative stress [51].

 C/EBP-*β* regulates several hepatic genes such as catalase and methionine adenosyltransferase 1a that are involved in regulation of oxidative stress [52, 53]. As oxidative stress plays an essential role in the pathogenesis of ALD, it is reasonable to argue that increased miR-155 observed in hepatocytes of alcohol-fed mice might contribute to increased oxidative stress via the regulation of genes involved in oxidative stress pathways. However, further studies are needed to prove this speculation in ALD.

 More recently, miR-155 has been demonstrated to play a role in antiviral immunity against HBV infection in human hepatoma cells, HepG2 [54]. Overexpression of miR-155 resulted in decreased SOCS1, which enhances STAT 1 and 3 phosphorylation [54]. Increased expression of several interferon-inducible antiviral genes was observed after ectopic expression of miR-155 in human hepatoma cells [54]. It was concluded that miR-155 acts as a positive regulator of JAK/STAT signaling via targeting SOCS1 and hence increases the expression of some IFN-inducible antiviral genes such as ISG15 and MxA [54].

 In this study, we also found decreased miR-125b in hepatocytes of alcohol-fed mice (Figure 2). Downregulation of miR-125b has been reported in HCC and is associated with increased placenta growth factor (PIGF) [55]. However, the physiological relevance of miR-125b downregulation in ALD has yet to be explored.

### 2.8. Increase of miR-155 and miR-132 in Kupffer Cells of Alcohol-Fed Mice

**Figure 3:**
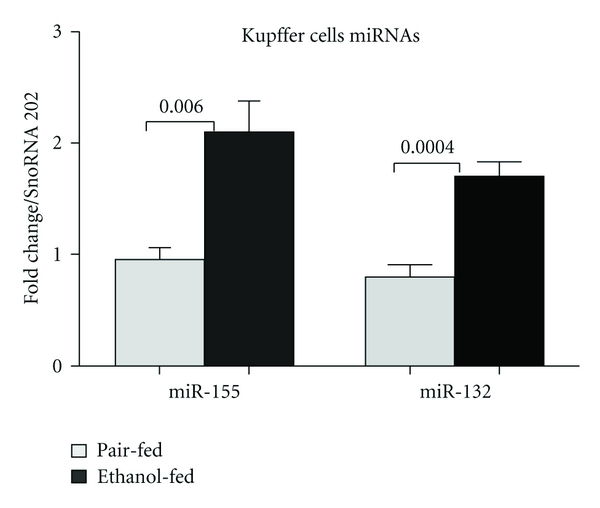
Upregulation of miR-155 and -132 in Kupffer cells of alcohol-fed mice. Kupffer cells were isolated either from pair-fed or alcohol-fed mice (*n* = 12–14/group, cells from two mice were pooled) after 4 weeks of feeding. Cells were plated and after 2 h of incubation nonadherent cells were removed. Fresh medium was added to the adherent, and they were rested overnight. Next day, cells were washed with PBS and lysed with QIAzole (Qiagen). Total RNA isolated using the miRNeasy kit was used quantify miRNA as described above. Data represent mean values ± S.E.M. Statistical significance was determined using T-test (two-tailed).

Next, we examined the expression of miRNAs in KCs of alcohol-fed mice. KCs were isolated as described earlier [34]. We focused our study on miR-155 and miR-132, as the expression of these miRNAs was increased in the livers after alcohol feeding (Figure 1). Interestingly, we not only observed a significant increase in miR-155, which was consistent with our previous report [34], but also induction of miR-132 in the KCs of alcohol-fed mice (Figure 3).

 Induction of miR-132 in alcohol-fed mice is interesting, as the role of this miRNA in innate immunity is not much appreciated. Recently, miR-132 was shown to play a role in the innate viral response, where it targets p300, a transcriptional coactivator [40]. Chronic alcohol use predisposes individuals to infections (bacterial and viral), and the induction of miR-132 after alcohol feeding is of great interest. We also found a modest increase of miR-132 in hepatocytes of alcohol-fed mice. The physiological relevance of miR-132 in the alcohol-mediated immune response is currently under investigation.

 Collectively, our results indicate an increase in miR-155 in different cell populations of the liver after alcohol feeding. It is most likely that the induction of miR-155 we observed in hepatocytes of alcohol-fed mice is involved in both LPS and oxidative stress signaling pathways, and hence contributes to the progression of ALD.

## 3. Conclusions

Our understanding of the role of miRNAs in liver disease is expanding, and the current studies suggest that miRNAs play a crucial role in alcoholic liver disease. However, in-depth understanding of association of miRNAs with their cell-specific roles in ALD is yet to be explored. We not only showed induction of miR-155, but also miR-132 in the livers and KCs of alcohol-fed mice. Furthermore, an increase in miR-155 was also observed in hepatocytes of alcohol-fed mice. The cell-specific effect of these miRNAs in ALD deserves further investigation.
